# Combining intra- and intermolecular charge-transfer: a new strategy towards molecular ferromagnets and multiferroics

**DOI:** 10.1038/srep19682

**Published:** 2016-01-21

**Authors:** Francesco Di Maiolo, Cristina Sissa, Anna Painelli

**Affiliations:** 1 Dipartimento di Chimica, Università di Parma, Parco Area delle Scienze 17/A, 43124, Parma, Italy

## Abstract

Organic ferroelectric materials are currently a hot research topic, with mixed stack charge transfer crystals playing a prominent role with their large, electronic-in-origin polarization and the possibility to tune the transition temperature down to the quantum limit and/or to drive the ferroelectric transition via an optical stimulus. By contrast, and in spite of an impressive research effort, organic ferromagnets are rare and characterized by very low transition temperatures. Coexisting magnetic and electric orders in multiferroics offer the possibility to control magnetic (electric) properties by an applied electric (magnetic) field with impressive technological potential. Only few examples of multiferroics are known today, based on inorganics materials. Here we demonstrate that, by decorating mixed stack charge transfer crystals with organic radicals, a new family of robust molecular ferromagnets can be designed, stable up to ambient temperature, and with a clear tendency towards multiferroic behaviour.

Electrical and magnetic properties of multiferroics can be manipulated by magnetic and electric fields, opening the way to intriguing technological applications^1^,^2^,^3^,^4^. Current research on multiferroics is mainly focused on inorganic materials, such as perovskite oxides (ABO_3_), whose physics is fairly well understood and whose intrinsic limitations are emerging^3^. Molecular multiferroics offer additional advantages, including wide availability and low-cost of raw materials, easy tailorability, mechanical flexibility and low weight. Recent papers discussing molecular multiferroics address coexisting ferroelectric and antiferromagnetic orders^5^,^6^. Indeed, in spite of long-lasting efforts^7^, just few examples of molecular ferromagnets are known, with extremely low transition temperatures^8^,^9^,^10^. We propose a novel strategy towards molecular multiferroics with coexisting ferroelectric (FE) and ferromagnetic (FM) orders, based on charge-transfer (CT) crystals with a mixed stack (MS) motif, a well-known family of ferroelectric materials^11^, decorated with organic radicals.

In MS-CT crystals electron donor (D) and acceptor (A) molecules alternate in 1D stacks, as illustrated in Fig. 1a for TTF-CA. Delocalized electrons along the stack lead to fractional charges …D^ρ+^ A^ρ−^D^ρ+^ A^ρ−^… and interesting properties^12^,^13^. Depending on the D and A strength, systems with a largely neutral (N, ρ < 0.5) or ionic (I, ρ > 0.5) ground state are formed and some of them, including TTF-CA, can be driven from N to I by decreasing temperature or increasing pressure^12^. The rich physics of MS-CT crystals is governed by strongly correlated electrons, delocalized over a 1D soft lattice, and is well described by a modified Hubbard model accounting for electron-phonon coupling^13^,^14^,^15^. The I stack is unstable toward dimerization^14^, leading to potentially FE states. Ferroelectricity was demonstrated in the I phase of TTF-CA^16^, and MS-CT salts represent one of the most promising families of organic FE^11^,^17^,^18^. In the ρ → 1 limit, spins on adjacent radical cations D^+•^ and anions A^−•^ are antiferromagnetically coupled. The proximity of a ferroelectric and an antiferromagnetic phase lead to an intriguing suggestion of multiferroicity^5^, and TTF-BA, a MS-CT crystal with ρ~1, was recently proposed as a magnetically controllable organic ferroelectric^19^.

Intramolecular CT governs the physics of D-π-A molecules, where D and A groups are bound by a π-conjugated bridge. Relevant structures are of interest for a variety of applications, including bio-imaging, sensing, solar cells, OLED, etc.^20^. In weakly-bound D-π-A dyads, bistability is observed when neutral and charge-separated (zwitterionic) states are close in energy^21^. Recently, a D-π-R^•^ dyad was synthesized where D is a TTF-derivative, and the PTM radical (R^•^) represents the A site^22^, as shown in Fig. 1b. This dyad turns zwitterionic (D^+•^-π-R^−^) in polar solvents, and, due to the tendency of ionized TTFs to self-aggregate, it forms dimers whose magnetic properties are dominated by a subtle interplay between inter and intramolecular CT^22^,^23^,^24^. Stimulated by these results, we propose a new strategy to design stable organic ferromagnets and prospective multiferroics, combining the FE properties of MS-CT crystals with the magnetic properties of D-π-R^•^ dyads.

## Results and Discussion

### The model

**Figure 1:**
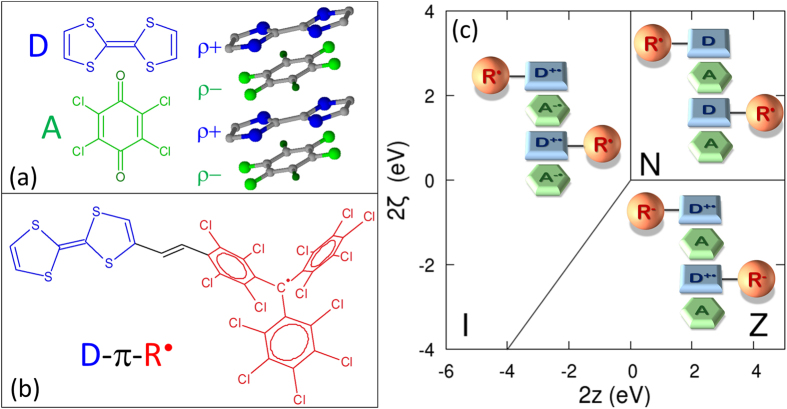
(**a**) A sketch of the TTF-CA stack, as a typical representative of MS-CT crystal, and the chemical structure of TTF and CA molecules, as typical D and A species, drawn in blue and green, respectively. (**b**) The chemical structure of the TTF-PTM molecule, as a representative of a D-π- R^•^ species. (**c**) The phase diagram showing the three dominant charge distributions in the (2*z*, 2ζ) plane.

Consider a molecular crystal, similar in structure to an MS-CT crystal (TTF-CA in Fig. 1a is a typical example), but with D substituted by a D-π-R^•^ dyad. Depending on the relative strength of D and A, and on the acceptor strength of R^•^, the three electrons present on each cell (two from D and one from R^•^) can be distributed in different ways, as sketched in Fig. 1c. In the most interesting situation, R^•^ maintains its unpaired electron while an almost full CT occurs from D to A. In this regime, in analogy with MS-CT salts, antiferromagnetic interactions between spins on adjacent D^+•^ and A^−•^ sites, combined with the antiferromagnetic interaction between spins on D^+•^ and R^•^, are expected to lead to a ferromagnetic locking of spins on R^•^ residues along the chain. In the same region of parameter space, the intrinsic instability of MS-CT crystals may promote dimerization and hence ferroelectricity, suggesting multiferroic behaviour.

We introduce a quantum-cell model for radical-decorated MS-CT crystals that integrates the modified Hubbard model of MS-CT salts^13^,^14^,^15^ with essential-state models of D-π-R^•^ dyads^21^,^23^,^24^. The decorated Hubbard model for a 1D chain reads:


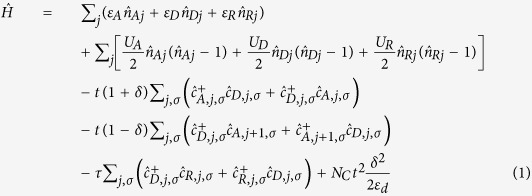


where *j* runs on the *N*_*c*_ cells, 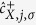 and 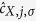 create and annihilate, respectively, an electron with spin σ on the X = A, D, R site of the *j*-th cell, and 
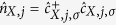
 is the corresponding number operator. The first term in the Hamiltonian accounts for on-site energies, ε_X_, and the second term for the repulsion, *U*_X_, between two electrons on the same site. The two subsequent terms describe the hopping of electrons between adjacent sites along the chain, with hopping integrals alternating between *t*(1 + δ) and *t*(1 − δ) to account for dimerization. The intramolecular hopping in the D-π-R^•^ dyad is described by τ. Finally the last term accounts for the dimerization elastic energy, with ε_d_ measuring the lattice relaxation energy^13^,^14^,^15^.

The total number of relevant model parameters can be reduced on physical basis. Specifically, states including A^2−^ or R^+^ sites are expected to marginally contribute to the ground state in view of their large energy. We therefore set *U*_R_, *U*_A_ and (ε_D_ − ε_R_) to infinity, as to impose infinite energy to all states with A^2−^ or R^+^ sites. Accordingly, the only relevant parameters are *U*_D_, 2ζ = ε_R_ − ε_D_ + *U*_R_ − *U*_D_, the energy required for the D-π-R^•^ → D^+•^-π-R^−^ process, and 2*z* = ε_A_ − ε_D_ − *U*_D_, the energy of the DA → D^+•^A^−•^ process. (see Fig S1 in the supporting information). This infinite correlation limit is in line with the description of D-π-R^•^ dyads based on just two essential states^21^,^23^,^24^, D-π-R^•^ and D^+•^-π-R^−^, and with the reduced basis approximation for MS-CT stacks^12^,^13^,^14^,^15^, where states including either A^2−^ or D^2+^ sites are excluded. Indeed we maintain D^2+^ states accessible: they are in fact instrumental to obtain sizable antiferromagnetic interactions in the D^+•^-π-R^•^ dyad. In the following we discuss results obtained setting *U*_D_ = 10 eV on a wide region of 2*z* and 2ζ plane; results for different *U*_D_ are shown in the supporting information.

We adopt the real-space basis, made up by the electronic configurations obtained distributing the electrons on the site-orbitals. Relevant energies are easily calculated in the (2*z*, 2ζ) plane, that can be partitioned in three regions, depending on the charge distribution in the dominant (lowest energy) configuration, as shown in Fig. 1c. In the positive 2z and 2ζ quadrant the system is dominated by neutral (N) states, with neutral D, A and R^•^ sites. The spins on R^•^ sites do not interact, leading to a paramagnetic phase. For negative 2ζ and 2*z* > 2ζ, intramolecular CT is favoured, with neutral A, but zwitterionic D^+•^-π-R^−^ dyads (phase Z). The unpaired spins on D^+•^ sites marginally interact, leading again to a paramagnetic phase. More interesting is the phase stabilized at negative 2z and 2ζ > 2z, where R^•^ maintains its unpaired electron, while one electron is transferred from D to A, leading to a phase analogous to the ionic phase (I) of MS-CT crystals^12^,^13^,^14^,^15^.

This last phase is particularly important for our aims, and is intriguing in many respects. Much as the I phase of MS-CT crystals, this phase corresponds to a Mott-insulator and only survives in the strong correlation limit, its width narrowing with decreasing correlations^25^,^26^. Accordingly, it cannot be captured within the Hartree-Fock approximation or mean-field treatment of electronic correlations^27^. Therefore we make resort to exact diagonalization techniques, to obtain fully correlated eigenstates of the single-chain Hamiltonian in equation (1) expressed in the real-space basis for finite size chains with periodic boundary conditions and an even number of cells, up to *N*_c_ = 6 (18 sites).

### Ferromagnetism

**Figure 2:**
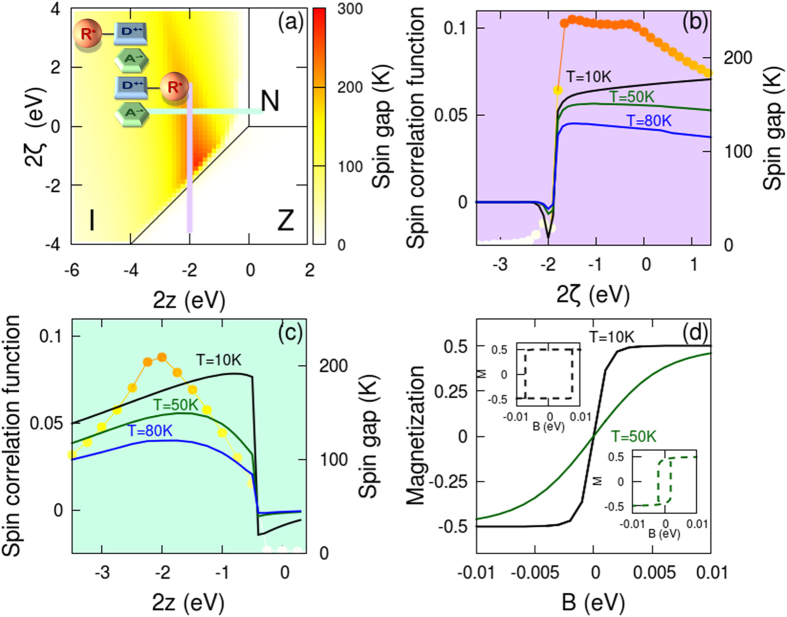
(**a**) The spin gap (energy of the lowest singlet eigenstate minus the energy of the lowest eigenstate in the *S* = *N*_C_/2 subspace) as a function of 2*z* and 2ζ, superimposed with the black lines marking the boundaries among the three phases. The spin gap vanishes in the paramagnetic N and Z phases, while it is positive and large (up to 300 K) in the I phase, confirming ferromagnetic behaviour. (**b**) The spin gap (dots) and the spin correlation functions (black, green and blue lines refer to different temperatures) on adjacent R^•^ radicals calculated vs 2ζ at fixed 2*z* = −2.0 eV, i.e. along the purple vertical line in panel (**a**). (**c**) The same as (**b**), but results are shown as a function of 2*z* at fixed 2ζ = 0.5 eV, i.e. along the green horizontal line in panel (**a**). (**d**) Evolution of the magnetization vs an applied magnetic field, calculated at 2*z* = −2.0 eV and 2ζ = 0.5 eV, corresponding to the crossing point of the purple and green lines in panel (**a**), for two different temperatures. The two insets show the same results calculated introducing a small interchain interaction, α = 0.005 eV.

To demonstrate FM interactions in the I phase, Fig. 2 reports results for a non-dimerized chain (δ = 0) with the intermolecular hopping integral set to the value characteristic of TTF-CA, t = 0.21 eV^13^,^14^,^15^,^28^, and the intramolecular hopping integral fixed as τ = 0.4 eV. This comparatively large value, definitely larger than the current estimate for the TTF-PTM dyad^23^,^24^, is needed to stabilize the FM phase, as shown in the supporting information where results obtained for different model parameters are collected. Figure 2a shows the energy gap between the lowest singlet (total spin S = 0) state and the lowest state with S = N_c_/2 state, superimposed with the phase diagram from Fig. 1c. The energy gap vanishes in the N and Z regions of the phase diagram, corresponding to paramagnetic phases, but it is finite and positive in the I region, fully supporting FM behaviour. Specifically, a large gap, up to 300 K, is observed in a wide portion of the phase diagram, suggesting stable FM up to ambient temperature.

Figure 2c shows the evolution against 2*z* of the spin gap calculated at 2ζ = 0.5 eV (corresponding to the green horizontal line in Fig. 2a) as well as the 2*z*-dependence of the spin correlation function on adjacent R^•^ sites:


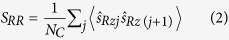


where 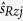 measures the z-component of the R^•^ spin in the *j*-th cell, and < > indicates the thermal average. *S*_RR_ values calculated at 10, 50 and 80 K amount to a sizable fraction of the limiting value, *S*_RR_ = 0.25, confirming FM order at finite temperature in the I phase. Similar results in panel (b), calculated at 2*z* = −2 eV for varying 2ζ (i.e. along the vertical purple line in Fig. 2a), further support this view. Data in Fig. 2, referring to *N*_*c*_ = 4, compare well with results for *N*_*c*_ = 6 (18 sites) in the supporting materials.

In the presence of an applied magnetic field, *B*, a new term enters the Hamiltonian:


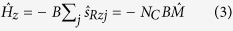


Figure 2d shows the *B*-dependence of the thermally averaged magnetization 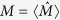 for a system with 2*z* = −2 eV and 2ζ = 0.5 eV. The steep slope of the curve at *B* = 0 supports again FM behaviour. Magnetic hysteresis loops require self-consistent interactions, as induced by interchain interactions. We therefore introduce a correction to the local magnetic field due to the polarization on the surrounding chains, substituting *B* in equation (3) with *B-αN*_*c*_*M*. Well pronounced FM loops are obtained imposing small α values (α = 0.005 eV, see insets of Fig. 2d).

### Ferroelectricity and Multiferroicity

**Figure 3:**
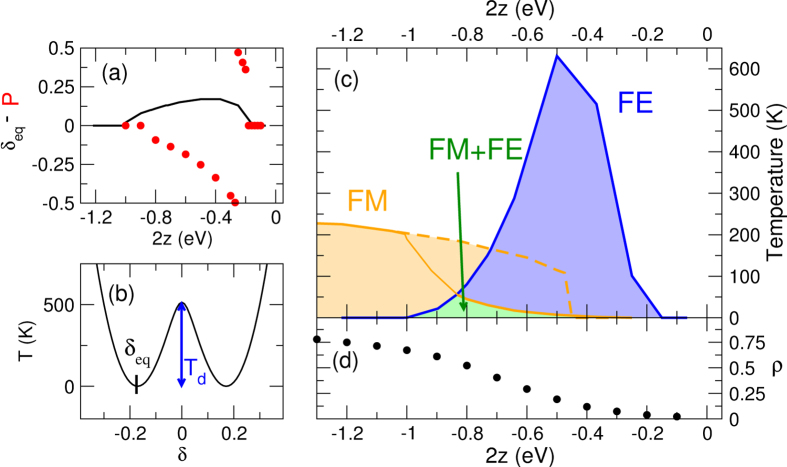
Results for a 6 cell system with the same model parameters as described in the text, and 2ζ = 0.5 eV and ε_d_ = 0.04 eV. The calculation of thermal averages for the dimensionless polarization, *P,* and for the ionicity, ρ, is extremely demanding, and relevant results (shown as dots in the figure) actually refer to a 4 cell system. (**a**) The evolution with 2*z* of the equilibrium dimerization amplitude (black) and of the 10 K electric polarization (red dots). (**b**) Ground state energy calculated for 2*z* = −0.368 eV as a function of δ: the double minimum is a signature of symmetry breaking. The minima locate the equilibrium dimerization amplitude, **δ**_eq_, and the barrier height offers an estimate of the dimerization temperature, *T*_d_. (**c**) The phase diagram: the orange area (*T* < *T*_M_) defines the region of stability of the FM phase, the blue region (*T* < *T*_d_) the region of stability of the dimerized, FE, phase. The green region defines the region of stability of the multiferroic phase. The dashed line shows the *T*_M_ temperature calculated for a system with **δ**_eq_ = 0. (**d**) Ionicity calculated as a function of 2*z* at 10 K; ρ is close to 1 in the ionic phase and becomes zero in the neutral one.

Having proved that in the I phase the unpaired spins on R^•^ sites experience a strong FM coupling, we now turn attention to FE interactions, as induced by stack dimerization. In the I phase MS-CT crystals undergo a dimerization transition, following a generalized-Peierls mechanism^12^,^13^,^14^,^15^,^28^. In the close proximity of the N-I interface, where the system is in a marginally metallic state, a Peierls mechanism governs the dimerization. Large dimerization energies, associated with charge degrees of freedom, lead to large transition temperatures and dimerization amplitudes (δ ~ 0.1 − 0.2). In the ρ → 1 limit, where the system maps into a Heisenberg antiferromagnet, a spin-Peierls mechanism drives the lattice instability. Relevant energies are much smaller, leading to low transition temperatures and small δ. These well-known results for MS-CT crystals survive in the decorated MS-CT system discussed in this study. Figure 3a shows the equilibrium δ calculated for the ground state, as a function of 2*z* for a system with *N*_*c*_ = 6, 2ζ = 0.5 eV and setting ε_d_ = 0.04 eV, in line with typical values for MS-CT crystals^13^,^14^,^15^. The dimerization amplitude vanishes deep in the N regime (positive 2z) as well as for large negative 2z, where ρ → 1. However, sizable dimerization amplitudes, δ ~ 0.15, are found close to the N-I interface.

A broken symmetry state is signalled by the presence of a double minimum in the ground state energy, as shown in Fig. 3b, where the barrier height offers a measure of *T*_d_, the dimerization temperature^14^,^28^. Figure 3c summarizes the thermal stability of FE and FM phases, calculated as a function of 2*z* for a system with *N*_*c*_ = 6, 2ζ = 0.5 eV and ε_d_ = 0.04 eV. In the high temperature region (white area in Fig. 3c) neither FE nor FM order is observed. The blue region below *T*_d_ marks the FE region, where the system spontaneously breaks the inversion symmetry, as demonstrated by finite δ values (Fig. 2a, black line).

To demonstrate FE behaviour, we calculate the dimensionless electric polarization, *P*, according to the following expression^26^,^29^:


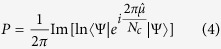


where Ψ is the ground state wavefunction, and 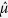 is the dimensionless dipole moment of the chain, defined in the supporting information. Red dots in Fig. 3a show relevant results calculated at 10 K (results for different temperature can be found in the supporting information). As the imaginary part of a complex logarithm, *P* is defined modulus the polarization quantum^26^ (equal to 1 for the dimensionless *P* in eq. 4). Accordingly, the principal logarithm (limited between ± π) is used in equation 4, limiting *P* to the relevant −0.5 < *P* ≤ 0.5 range. As it happens for MS-CT crystals^26^,^29^, the polarization changes sign at the N-I interface, an intriguing phenomenon that is due to the different nature of the two insulating N and I phases^26^. Quite interestingly, close to the interface, *P* attains the maximum theoretical value, *P* = ±0.5, corresponding to a very large dimensional polarization, *eσP* (where *e* is the electron charge and σ the density of chains per unit surface), estimated up to ~14 μC/cm^2^, using σ = 0.018 Å^−2^, as relevant for TTF-CA^30^. This value, corresponding to the maximum theoretical *P* value, is more than an order of magnitude larger than the polarization measured for TTF-BA^19^.

The orange region (*T* < *T*_M_) in Fig. 3c marks the FM phase discussed in the previous Section. Dimerization weakens FM correlations: indeed the spin gap calculated for the equilibrium dimerization is somewhat smaller than the value obtained for the non-dimerized stack (shown as a dashed line in Fig. 3c). However a sizable region (green in the figure) survives with coexisting FE and FM properties, pointing to multiferroic behaviour. The coexistence of the two orders is an obvious prerequisite for multiferroicity, but does not grant for their actual coupling. Indeed, at the single chain level the interaction between ferrolectricity and ferromagnetism is negligible with vanishing derivatives of the magnetization on an applied electric field or vanishing derivatives of the electrical polarization on an applied magnetic field. However the situation could change if a more detailed model of interchain interactions is introduced. But to build reliable models for interchain interactions specific crystal structure data are needed.

MS-CT crystals show a very rich physics, including temperature and pressure induced phase transitions^12^ that can be driven down to the quantum limit 31,32, multistability^33^, photoinduced phase transitions^34^, large dielectric constants^35^, etc. Several anomalies in vibrational spectra^15^ and diffuse X-ray scattering^36^ of MS-CT crystals demonstrate large electron-phonon coupling. The FE behaviour of MS-CT crystals is well understood as resulting from the dimerization instability of the stack in the I phase. Antiferromagnetic interactions between unpaired spins on D^+•^ and A^−•^ sites are prominent in the same phase. Here we exploit the known physics of MS-CT crystals to propose a new family of crystals where stable radicals are attached to D sites via a strong enough π-bridge as to drive antiferromagnetic interactions between spins on D^+•^ and R^•^ fragments. A subtle interplay between delocalized electrons along the DA stack and intramolecular CT in the D-π-R^•^ units takes place, and the already impressive physics of MS-CT crystals is further enriched in these new systems. Based on a novel quantum-cell Hamiltonian, we demonstrate that the decorated MS-CT crystal supports ferromagnetic interactions in a large region of the parameter space, leading to a FM state stable up to ambient temperature that can be driven towards a potentially multiferroic phase at low temperature.

Designing and synthesizing relevant materials is far from trivial. To guide the discussion, Fig. 3d shows the 2*z* dependence of 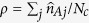, the charge transferred from D to A (in the relevant region the charge on R^•^ is zero). The FM phase is stabilized far in the I region, where ρ is well above 0.5. Several largely ionic mixed stack systems are known^12^, based on strong donors (TTF, TMPD, M_2_P, etc.) and strong acceptors (BA, TCNQ, TCNQF_4_, etc.). A few families of stable and persistent organic radicals are known^9^,^10^ that could be attached to an electron-donor to lead to the (D–π–R^•^)A structure schematized in Fig. 1c. Indeed R^•^ -decorated TTF or TTF derivatives have already been synthesized, with R^•^ = PTM^22^ (as schematically shown in Fig. 1b), 6OP (2,5-di-*tert*-butyl-6-oxophenalenoxyl-radical)^37^, or TEMPO (tetramethylpiperidin-N-oxyl radical)^10^. Unfortunately, the intramolecular D–R^•^ conjugation is not very effective in these systems. TTF-PTM derivatives are in this respect the most promising systems, but the estimated τ = 0.1 eV^23^ would lead to a FM region with low transition temperatures in a large region of the phase diagram. Strategies to improve the D–π–R^•^ conjugation may involve the rigidification of the bridge and the insertion of specific substituents as to better align the energies of the frontier orbitals of the D, π and R^•^ residues. Offering theoretical guidance on this issue is however difficult: a reliable first principle estimate of τ would require calculations on ground and low-lying excited states of large radicals with strong CT character, where high-quality ab-initio calculations are impractical and TD-DFT is poorly reliable.

To drive the system into the FE-FM coexistence region, systems with intermediate ionicity, 0.3 < ρ < 0.7, are in demand. Just few mixed stack crystals with intermediate ionicity are known, and they are typically unstable with respect to dimerization (so that they are potentially ferroelectric). Again TTF and TTF-related molecules seem to be promising D species, combined with CA, BA (bromanyl) and mixed chloro/bromo-substituted quinones. As discussed above for the FM phase, once the D molecule is selected, decorating it through a conjugating bridge to a stable radical is a challenging task and care has to be taken, since the insertion of a new group may alter the crystal structure with respect to the parent mixed stack crystal. Overall, the chemical requirements are stringent, but the tool-box of molecular and supramolecular chemists is powerful enough to successfully face the challenge towards the synthesis of stable molecular ferromagnets and multiferroics.

## Methods

Fully correlated eigenstates of the Hamiltonian in equation (1) are obtained via real-space diagonalization on finite size chains with periodic boundary conditions and an even number of cells, up to *N*_*c*_ = 6 (18 sites), working in subspaces with different total *S*_z_^38^. Basis states are defined assigning the electronic occupancy of the frontier spin-orbitals on D, A and R^•^ sites. A reduced basis approximation is adopted, disregarding states with A^2−^ and R^+^ sites. Singlet, triplet, quintet… states are recognized by searching for degenerate states in subspaces with different *S*_z_. The dimension of the basis increases rapidly with *N*_*c*_: the *S*_z_ = 0 subspace has 97320 states for *N*_*c*_ = 4 and more than 85 million states for *N*_*c*_ = 6. To make the problem numerically tractable we reduce the dimension of the matrices implementing translational symmetry and diagonalizing the problem in the subspaces with different wavevectors^38^. Thermal averaged calculations involve complex symmetry-subspaces and become very time-consuming for *N*_*c*_ = 6. Sparse Hamiltonian matrices were stored using the CSR format in the 3-Array Variation. Very efficient diagonalization routines from ARPACK^39^ were exploited for real symmetric matrices on a 16 GB computer with 4 physical cores each dealing with two threads at 3.6 GHz and on the supercomputer GALILEO at CINECA, using one computer node (128 GB) with 2 octa-core at 2.4 GHz. In order to diagonalize big complex Hermitian matrices, we used routines from JADAMILU package^40^ on the same computers.

According to equation (3), the magnetic field entering the Hamiltonian has energy units. To transform to Tesla unit it must be divided by *g*_e_μ_B_, where *g*_e_ is the electron g-factor and μ_B_ is the Bohr magneton. Accordingly, the *B* values ranging from −0.01 to 0.01 eV in Fig. 2d correspond to a range of *B* values approximately running from −86 to 86 Tesla.

## Additional Information

**How to cite this article**: Di Maiolo, F. *et al.* Combining intra- and intermolecular charge-transfer: a new strategy towards molecular ferromagnets and multiferroics. *Sci. Rep.* **6**, 19682; doi: 10.1038/srep19682 (2016).

## Supplementary Material

Supplementary Information
