# Supplementary material for: Combining intra- and intermolecular charge-transfer: a new strategy towards molecular ferromagnets and multiferroics
Source: Sci Rep. 2016 Jan 21;6:19682. doi: 10.1038/srep19682 (PMC4726343; doi:10.1038/srep19682)
Supplement: Supplementary Information [file srep19682-s1.pdf]

Combining intra- and intermolecular charge-transfer: a new strategy towards molecular ferromagnets and multiferroics

*Francesco Di Maiolo, Cristina Sissa, Anna Painelli**

Dipartimento di Chimica, Università di Parma, Parco Area delle Scienze 17/A, 43124, Parma Italy

Supporting Information

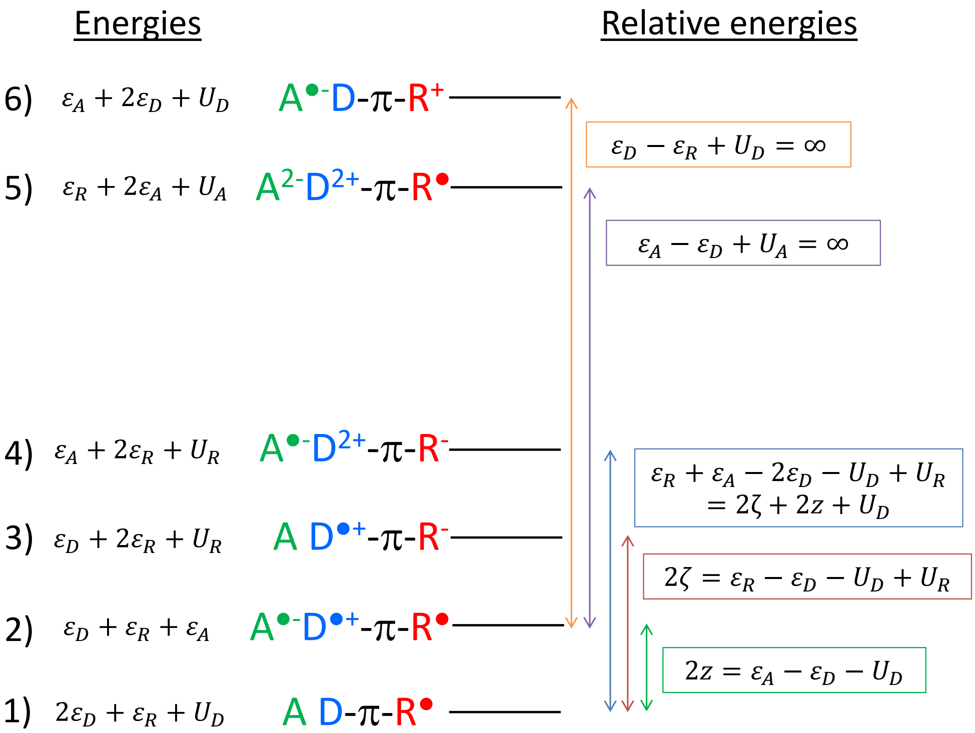

Figure S1: The reduced basis approximation, discussed based on the charge configuration for single cell. States 1-4 have finite energies, fully defined by the three parameters, $2z$, 2ζ and U_D. The energy of state 5, having doubly charged A, is set to infinity by setting $U_A \rightarrow \infty$. The energy of state 6, having a positively charged R site, is set to infinity by setting $(\varepsilon_D - \varepsilon_R) \rightarrow \infty$. The quantity 2ζ stays finite as the difference of two infinite quantities, U_R and $(\varepsilon_D - \varepsilon_R)$.

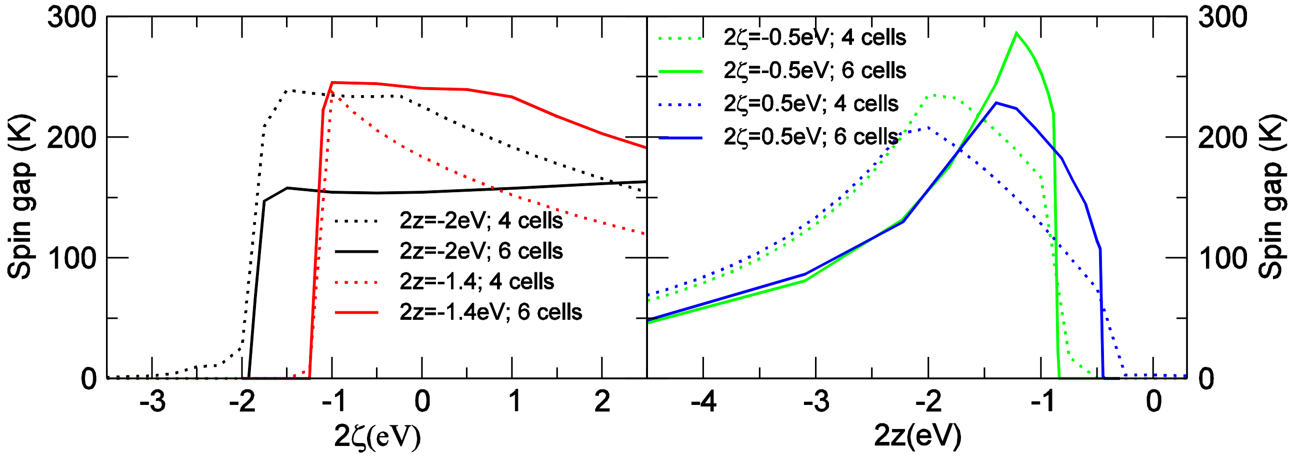

Figure S2: Finite size effects on the spin gap. The spin gap is reported as a function of 2ζ (left) and $2z$ (right), for a 6-cell system (18 sites), and different values of $2z$ (left) and 2ζ [illegible]). For comparison, dotted lines show results calculated for 4-cell (12 sites). All results refer to a system with τ=0.4eV, *t*=0.21 eV and U_D=10 eV.

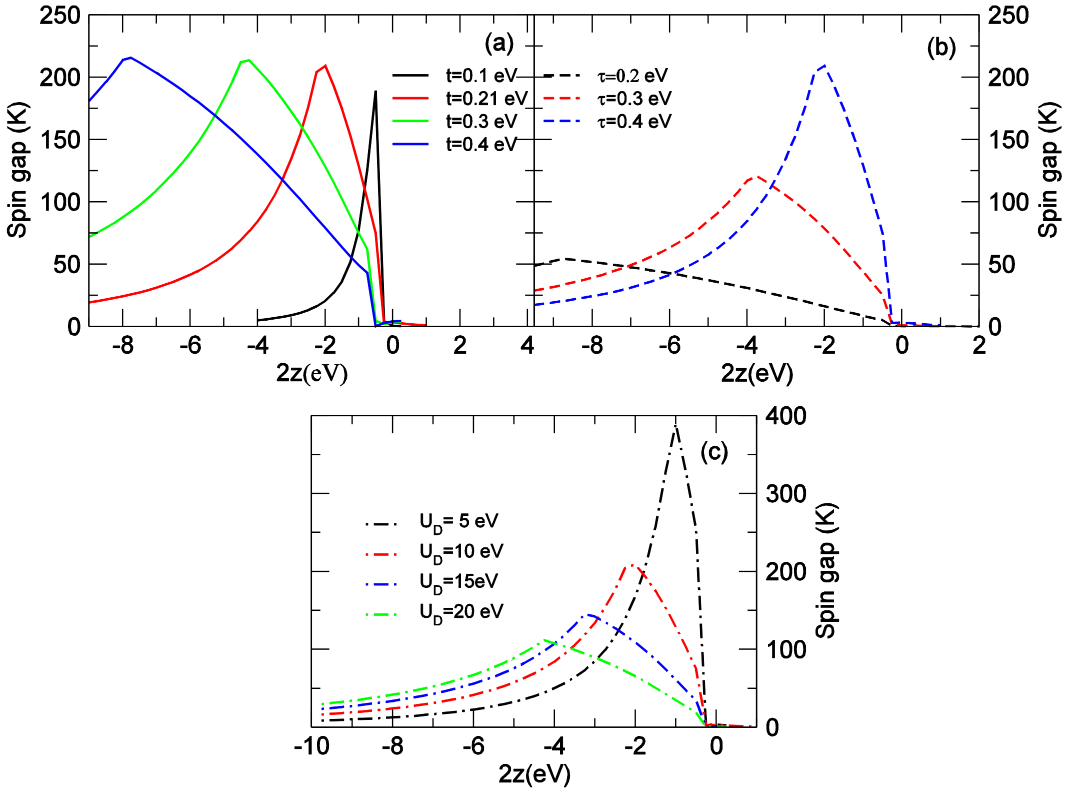

Figure S3: The effects of varying model parameters. (a) The spin gap calculated as a function of $2z$ for a 4-site chain with 2ζ=0.5eV, τ=0.4 eV, U_D=10 eV, and variable *t*. (b) Spin gap calculated as a function of $2z$ for a 4-cell chain with 2ζ=0.5eV, *t*=0.21eV, U_D=10 eV, and variable τ. (c) Spin gap calculated as a function of $2z$ for a 4-site chain with 2ζ=0.5eV, *t*=0.21eV, τ=0.4 eV and variable U_D.

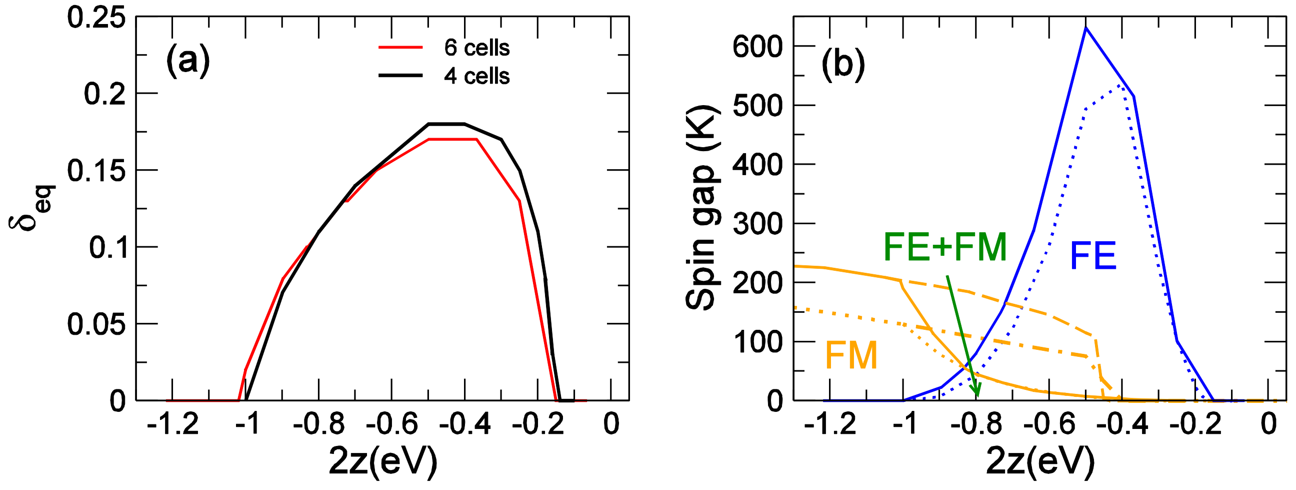

Figure S4: (a) Finite-size effects on the dimerization amplitude. (b) Comparison of the phase diagram in Fig. 3c (calculated for N_c=6) with the results obtained for N_c =4, shown as dotted lines (the dash-dotted line shows the spin gap calculated for δ=0).

The dipole moment operator, $\hat{\mu}$, entering equation (4) in the main text, measures the dipole moment along the chain axis as[29]:

$$\hat{\mu} = \sum_{n=1}^{N_C}\left[\left(\hat{\rho}_R + \hat{\rho}_D\right)\left(2n - 1 + \frac{\delta}{2\tilde{\alpha}}\right) + \hat{\rho}_A\left(2n - \frac{\delta}{2\tilde{\alpha}}\right)\right]$$

where $\hat{\rho}_R = 1 - \hat{n}_R$, $\hat{\rho}_D = 2 - \hat{n}_D$ and $\hat{\rho}_A = -\hat{n}_A$ are the operators that measure the net charges on sites $R^{\bullet}$, D and A. The dimensionless dipole moment in the above equation is expressed in units with *ea*=1, where a is the D-A distance. The position of each site is defined in terms of the cell number, *n,* and of the dimerization amplitude δ. The prefactor of δ contains the dimensionless coupling constant $\tilde{\alpha} = \left(\frac{a}{t}\right)\sqrt{K\varepsilon_d}$, where K is the force constant of the dimerization mode. Full symbols in Fig. S4a show the total polarization calculated according to equation (4), setting $\tilde{\alpha} = 5$, as estimated for TTF-CA[29] (the same data as in Fig. 3a in the main text). In the same figure open symbols show the purely electronic contribution to the polarization, obtained neglecting the δ correction in the definition of $\hat{\mu}$ (i.e. setting δ=0 in the above equation). As expected,[29] the purely electronic polarization largely dominates the polarization.
Fig. S4b compares the total polarization calculated for different temperatures.

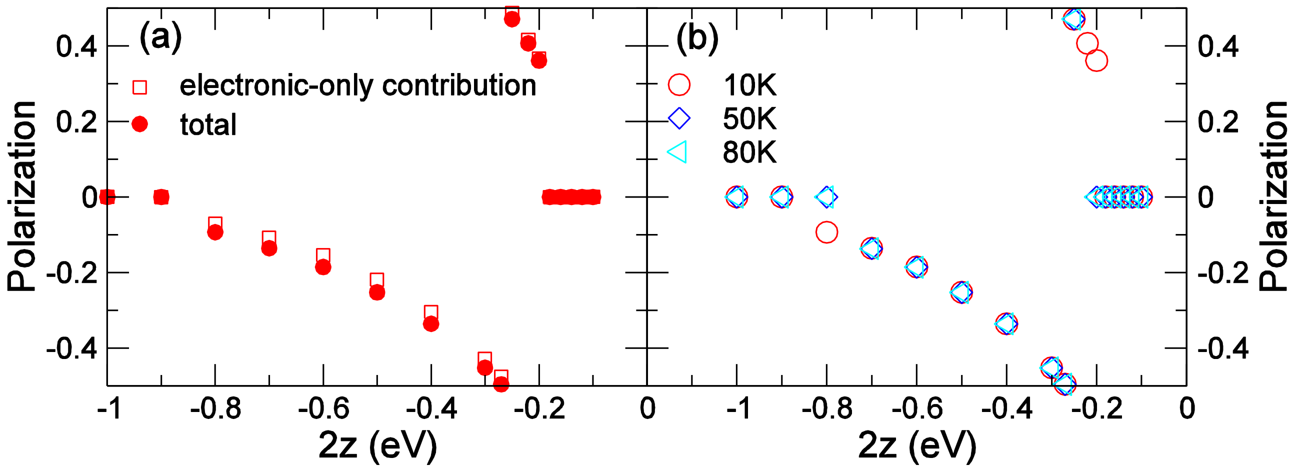

Figure S5: Results for a 4 cell system. (a) $2z$-dependence of the total polarization (i.e., both electronic and ionic contributions) and of the purely electronic contribution, calculated at 10K. (b) Total electronic polarization calculated for three different temperatures.
